# Fast Fingerprint Database Maintenance for Indoor Positioning Based on UGV SLAM

**DOI:** 10.3390/s150305311

**Published:** 2015-03-04

**Authors:** Jian Tang, Yuwei Chen, Liang Chen, Jingbin Liu, Juha Hyyppä, Antero Kukko, Harri Kaartinen, Hannu Hyyppä, Ruizhi Chen

**Affiliations:** 1 GNSS Research Center, Wuhan University, 129 Luoyu Road, Wuhan 430000, China; E-Mail: tangjian@whu.edu.cn; 2 Department of Remote Sensing and Photogrammetry, Finnish Geospatial Research Institute, Geodeetinrinne 2, Kirkkonummi FI-02431, Finland; E-Mails: jingbin.liu@nls.fi (J.L.); juha.hyyppa@nls.fi (J.H.); antero.kukko@nls.fi (A.K.); harri.kaartinen@nls.fi (H.K.); 3 Department of Navigation and Positioning, Finnish Geospatial Research Institute, Geodeetinrine 2, Kirkkonummi FI-02431, Finland; E-Mail: liang.chen@nls.fi; 4 Department of Real Estate, Planning and Geoinformatics, Aalto University, P.O. Box 11000, Espoo FI-00076, Finland; E-Mail: hannu.hyyppa@aalto.fi; 5 Conrad Blucher Institute of Surveying & Science, Texas A&M University at Corpus Christi, Corpus Christi, TX 77843-3577, USA; E-Mail: ruizhi.chen@tamucc.edu

**Keywords:** laser scanning, fingerprint database, indoor positioning, SLAM, SOP

## Abstract

Indoor positioning technology has become more and more important in the last two decades. Utilizing Received Signal Strength Indicator (RSSI) fingerprints of Signals of OPportunity (SOP) is a promising alternative navigation solution. However, as the RSSIs vary during operation due to their physical nature and are easily affected by the environmental change, one challenge of the indoor fingerprinting method is maintaining the RSSI fingerprint database in a timely and effective manner. In this paper, a solution for rapidly updating the fingerprint database is presented, based on a self-developed Unmanned Ground Vehicles (UGV) platform NAVIS. Several SOP sensors were installed on NAVIS for collecting indoor fingerprint information, including a digital compass collecting magnetic field intensity, a light sensor collecting light intensity, and a smartphone which collects the access point number and RSSIs of the pre-installed WiFi network. The NAVIS platform generates a map of the indoor environment and collects the SOPs during processing of the mapping, and then the SOP fingerprint database is interpolated and updated in real time. Field tests were carried out to evaluate the effectiveness and efficiency of the proposed method. The results showed that the fingerprint databases can be quickly created and updated with a higher sampling frequency (5Hz) and denser reference points compared with traditional methods, and the indoor map can be generated without prior information. Moreover, environmental changes could also be detected quickly for fingerprint indoor positioning.

## 1. Introduction

Indoor positioning and navigation systems have become increasingly significant with their development in terms of accuracy, reliability and availability in recent years. Utilizing Signals of Opportunity (SOP) is a promising alternative navigation means which may serve in GNSS-challenged environments, such as indoors [1]. Meanwhile, SOPs exist as non-navigation radio frequency signals around us, such as WiFi, Bluetooth, digital broadcasting signals, ZigBee, magnetic field, light, *etc.* [2,3,4,5,6,7]. Their patterns in the environment can become unique features for estimating location using the fingerprinting method.

Fingerprinting is a feasible technique for positioning using Received Signal Strength Index (RSSI) measurements. The basic idea of the fingerprinting method is to match a database to a particular fingerprint in the area at hand. The method operates in two phases: the training phase and the online positioning phase. In the training phase, the SOP map is created based on the reference points within the area of interest. The SOP map implicitly characterises RSSI positional relationships through the training measurements at the reference points with known coordinates. In the online positioning phase, the mobile device measures RSSI observations, and the positioning system utilises the SOP map to obtain a position estimate. The fingerprinting method has been widely discussed for indoor positioning, and various factors that affect fingerprinting are thoroughly summarised in [8]. Different fingerprinting algorithms are compared for indoor positioning with Wireless Local Area Networks (WLAN) in [7,9,10].

For fingerprint positioning, the traditional method of manually building a fingerprint database is usually labour intensive and time consuming, especially in a large mapping area with a high resolution of calibration points, which is required for storage in the database. Moreover, SOP signals are sensitive to environment change; for example, adding or removing a steel-made table in the office may totally distort the previous magnetic pattern. Rearranging the layout of a supermarket will disturb the distribution of the WiFi pattern significantly. This implies that the fingerprint database should be maintained timely according to environmental changes to guarantee its availability and accuracy by recalibration. Obviously, this maintenance is a high-cost labour [11]. The topic of sustaining freshness of the fingerprint database has already attracted the attention of researchers in the last few years. Rai *et al.* [12], Shen *et al.* [13] proposed an inertial positioning method based on the Pedestrian Dead Reckoning (PDR) of smartphone users to calibrate the WiFi Fingerprint database, which must model the walking mode of pedestrians. SmartSLAM [14] employs inertial tracing, a WiFi observation model and the Bayesian estimation method to construct the floor plan. FootSLAM [15] also utilises shoe-mounted inertial sensors to construct the indoor map. WiFi-SLAM [16] exploits a Gaussian process latent variable model to build WiFi signal strength maps and can acquire topographically correct connectivity graphs. However, most of the above methods rely greatly on the measurements of IMU, while current IMU manufacturing technology still restricts their applicability, and the embedded consuming-level IMUs in mobile devices might not guarantee its position estimation in complex indoor environments. Moreover, all aforementioned methods cannot collect and update spatial maps. Although Scholl *et al.* [17] and Lee *et al.* [18] also proposed a similar method, fewer SOP signals and no environmental variation are considered in their research.

In this study, we introduce a self-designed autonomous SLAM (simultaneous localization and mapping) robot platform NAVIS [19] by taking advantage of the feature with accurate positioning of the reference point and indoor mapping simultaneously. The objective is to carry out the SOP data collection for indoor positioning. Based on the platform, the indoor map can be built and updated simultaneously, which is important for navigation applications. The SLAM mapping algorithm calculates its accurate position of the robot platform. All SOP pattern including RSSI, light strength, magnetic field strength will be collected and updated with corresponding position. The positioning accuracy from mapping algorithm plays an important role to sustain the accuracy of the database. We compared the mapping results from mapping algorithm with terrestrial laser scanning (TLS) in feature-less environment and open data (Intel Seattle lab) in feature-rich environment as reference. We evaluated the accuracy of the proposed SLAM mapping algorithm and concluded that such method can be utilised for miscellaneous SOP fingerprint database maintenance of pedestrian indoor navigation in a quick manner.

The main contributions of this paper are included as follows: (1) a faster LiDAR-UGV based SLAM method for SOP collection is designed and tested with denser SOP sample points, higher sampling frequency and larger coverage area; (2) an accurate spatial map can be created and updated simultaneously with the proposed method, which can be utilised for indoor navigation; (3) the scalability of the system is evaluated from WiFi-only SOP source positioning to miscellaneous SOP source positioning, and the preliminary experiment proves the that miscellaneous SOP positioning method can enhance positioning accuracy by 19% percent compared with the WiFi-only solution with an un-optimised algorithm to offer a readily accessible solution for indoor positioning with higher availability. The rest of this paper is organised as follow: Section 2 describes the workflow of SOP fingerprint database maintenance using NAVIS. Section 3 discusses the field tests and the experimental results, and conclusions are drawn in Section 4.

## 2. SOP Fingerprint Database Maintenance Method using UGV SLAM

### 2.1. Method Overview

**Figure 1:**
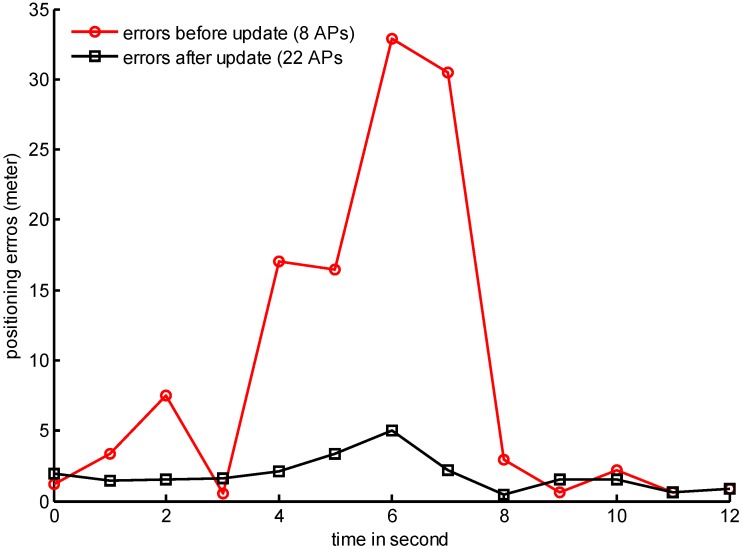
Comparison of positioning errors before/after the updated set of access points.

One of the challenges of the fingerprinting method is generating and maintaining the fingerprint database. SOP signals such as WiFi, Bluetooth, magnetic field, digital broadcasting signals, *etc.*, vary during operation due to their physical nature and are vulnerable to changes of the environment. The positioning results drift quickly if the fingerprint database cannot be updated in time, which results in the inapplicability of the fingerprinting method in reality. Figure 1 presents a practical sample tested by authors in a typical office environment. When the Access Point (AP) set changes from 22 APs to 8 APs in the third floor of the Finnish Geospatial research Institute (FGI) main office building, the positioning accuracy decreases from 1.87 m to 8.96 m with un-updated database. Traditional method manually builds a fingerprint database by measuring SOP pattern in a known reference point for a period time, for example, 30 s to 1 min, and the mean value is calculated as the fingerprint feature information. It is a labour intensive and time consuming task and difficult to update it. A readily accessible method for maintaining the fingerprint database is still not available, which restricts the applicability of the indoor navigation applications.

**Figure 2:**
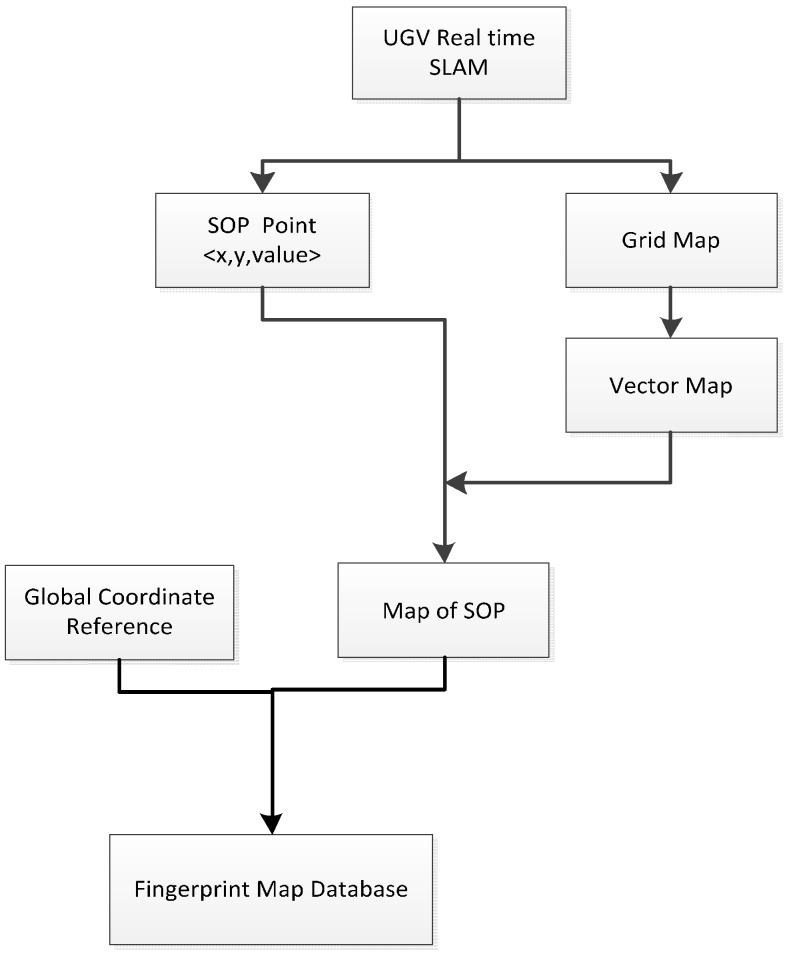
Workflow of SOP fingerprint database maintenance based on UGV SLAM.

SLAM technology may become an effective method for resolving such problems. Figure 2 shows the workflow of the fingerprint database maintenance method proposed in this paper. The self-developed NAVIS platform equipped with a LiDAR, a magnetic meter, a light strength sensor, and a smart phone runs through the unknown indoor environment. It generates an indoor grid map and collects the SOP signals simultaneously in real time along a trajectory. Mapping algorithm of SLAM calculates accurate positions of the moving platform as reference points. All SOP pattern including RSSI, light strength, magnetic field strength will be collected and updated with their corresponding reference point. The positioning accuracy from the mapping algorithm plays an important role for sustaining the accuracy of the database and defines the error envelope of the indoor positioning applications. The indoor grid map generated by the laser point cloud can be utilised for indoor navigation. Finally, the SOP fingerprint map database is interpolated within the vector indoor map and rectified to the global map coordinate reference for seamless outdoor-indoor navigation applications.

### 2.2. Real Time SLAM Based on “NAVIS”

**Figure 3:**
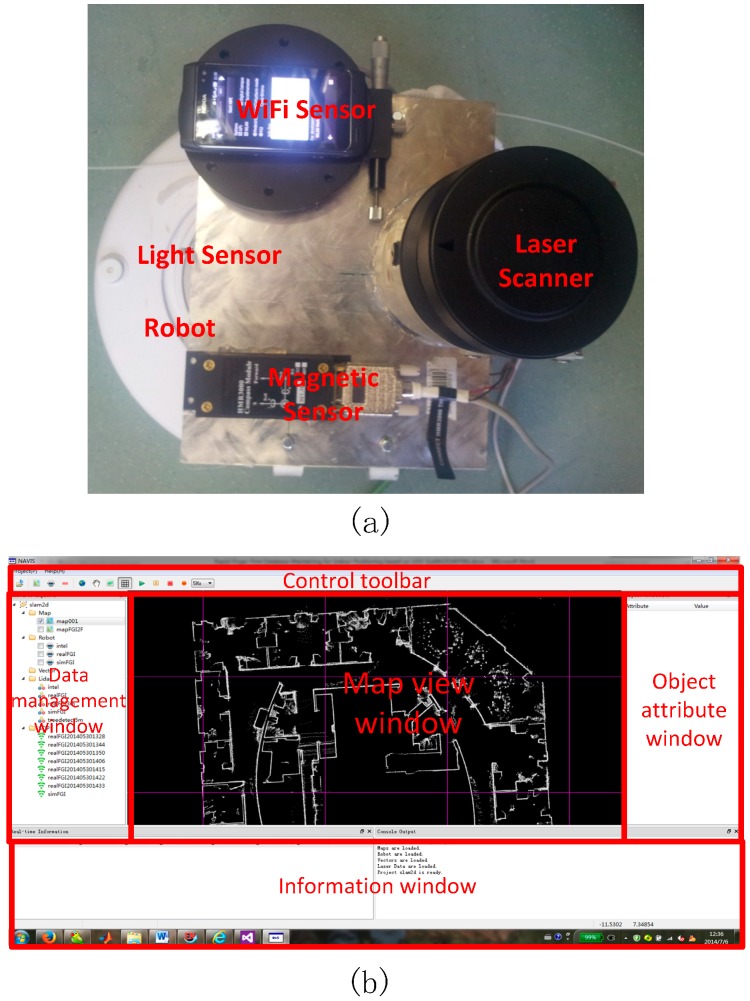
(**a**) The hardware platform of real time UGV SLAM NAVIS; (**b**) the data processing software of NAVIS.

The core of the proposed fast fingerprint database maintenance method is to obtain the accurate position of the SOP reference point as quickly as possible. A real-time 2D UGV with LiDAR-based SLAM technology is utilised for that purpose in this work. The scan-matching algorithm designed for the SLAM is called the Improved Maximum Likelihood Estimated (IMLE) based on a multi-resolution occupied grid map [19]. The NAVIS can achieve a positioning and mapping frequency of 5 Hz on an on-board computer with an average positioning accuracy of approximately RMS 10 cm. The NAVIS platform is based on: (1) an iRobot^®^ home vacuum-cleaning robot (see Figure 3a); (2) several SOP sensors including digital compass (HMR3000, Honeywell), light sensor (a self-developed module with a CdS photoresistor), and a WiFi sensor (a smartphone with self-developed RSSI collecting program); and (3) a SICK LMS150 laser scanner. The laser scanner has a field-of-view of 270° with 0.25° angular resolution and a scan frequency of 25 Hz, and the maximum effective range for the laser scanner is 50 m indoors. The laser scanner, the SOP sensors and the robot are all connected to the on-board computer through its Ethernet port for collecting laser scanning data and serial ports connections for SOP sensors. The LiDAR and all SOP sensors are powered by an external battery. Figure 3b shows the Graphic User Interface (GUI) of the NAVIS program, which is designed and implemented for data management, positioning and mapping. The SLAM mapping and positioning result are presented in the centre “map view window”, and data resources are organized in the data management window on the left.

### 2.3. Mapping Accuracy Evaluation

**Figure 4:**
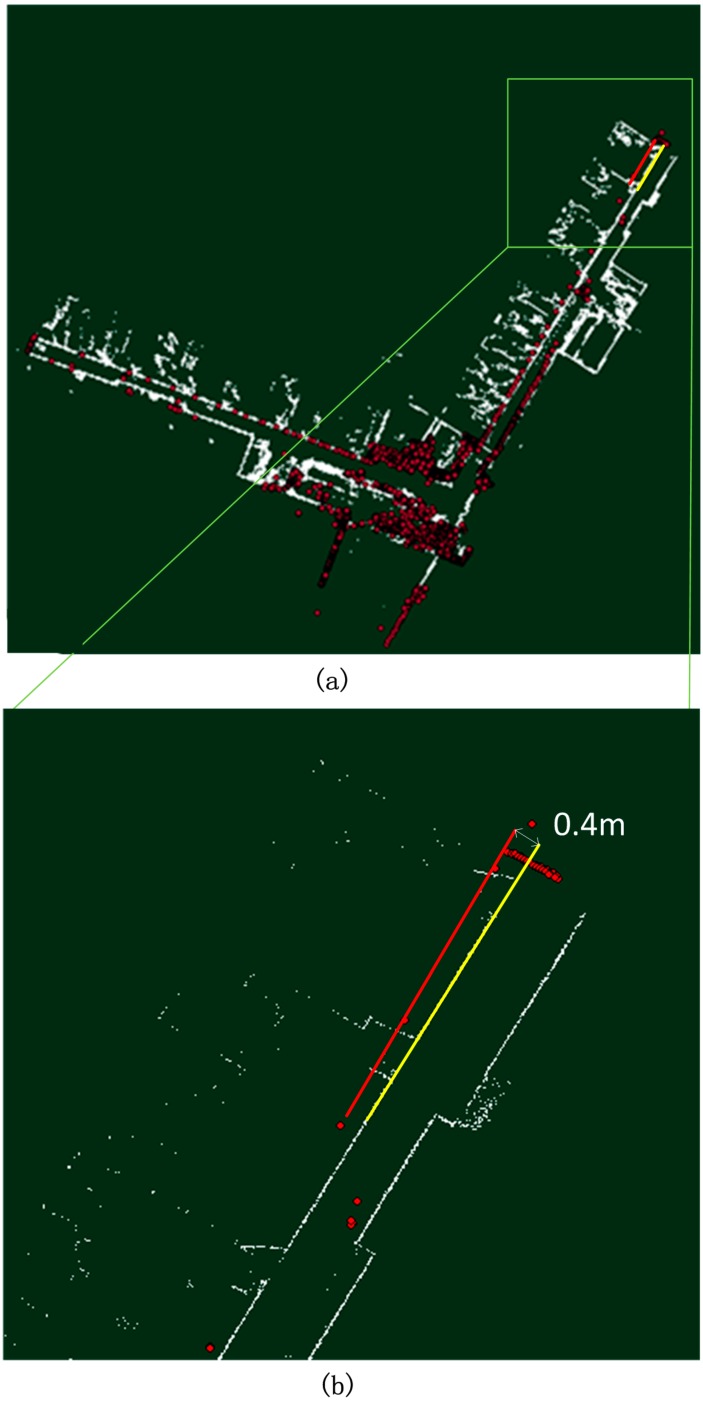
(**a**) The NAVIS mapping result of the FGI corridor compared with TLS; (**b**) map error details.

**Figure 5:**
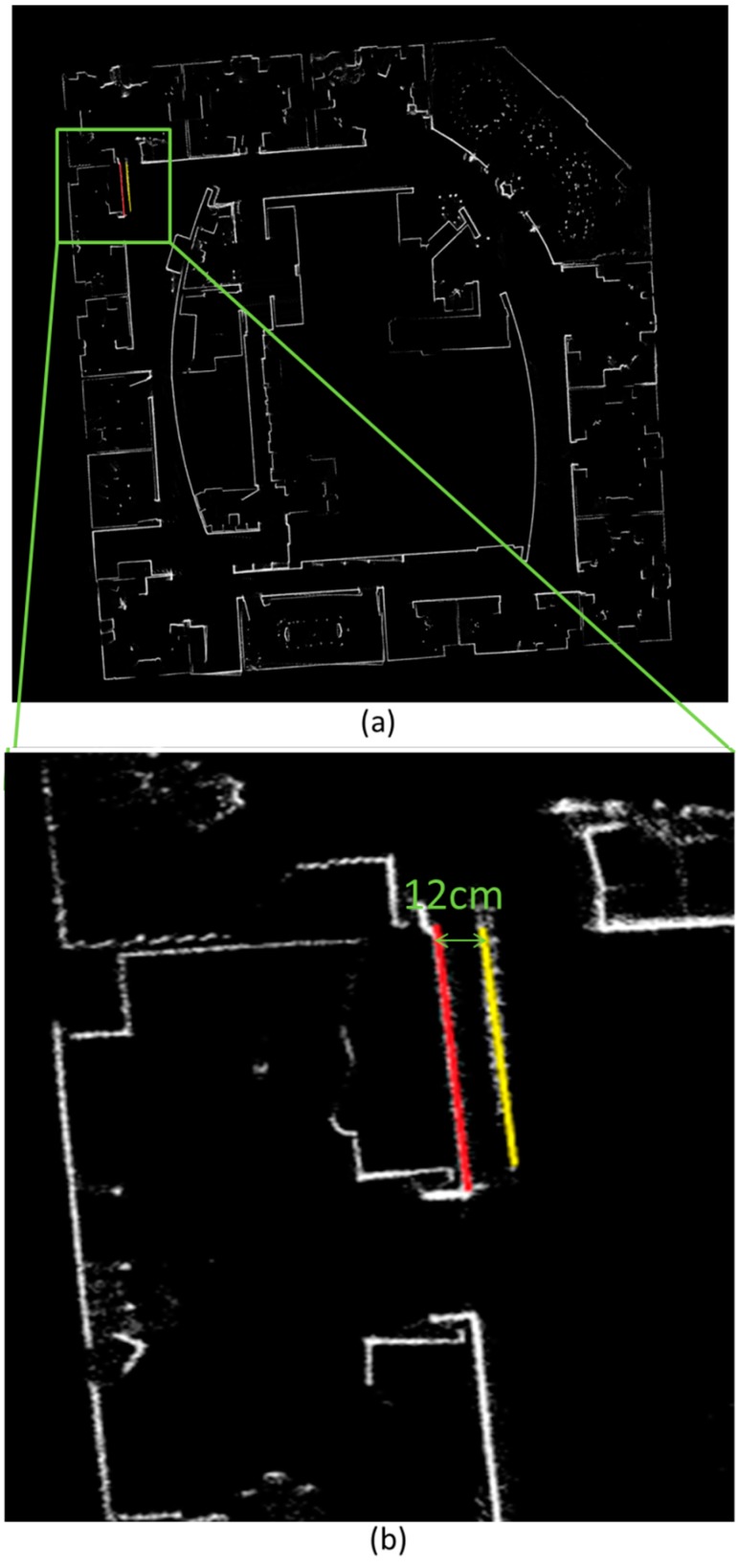
(**a**) Mapping error details of open dataset; (**b**) Mapping result of NAVIS tested with open dataset (Intel Research Lab-Seattle).

Figure 4 presents the mapping results of the mobile NAVIS platform of the third floor of the FGI main building compared with that of Terrestrial Laser Scanning (TLS) in which a laser scanner surveys the environment on a stationary tripod. The red dots are the TLS reference, and the white grid map is the result of NAVIS. The left (west) corridor is aligned and coincides well with the reference points, but there is a 0.4 m deviation at the end of the right (north-east) corridor illustrated as the red and yellow lines in the figure. There are several possible reasons for the deviation. The first is that the heading estimation resolution of NAVIS is 0.5°. This implies that the error introduced by quantisation of the heading estimation is 19.6 cm at the end of the right (east) corridor, which has a length of 45 m. The second is that the corridor turn in FGI is a feature-poor indoor environment with glass-made windows and handrails which do not reflect the laser pulse like a Lambertian object. Only the echoes from the steel window frame can be utilised for scan-matching processing. This low-feature environment results in the decrease of the heading estimation, and the positional error introduced by the heading estimation accumulates as the travelled distance increases. We also tested NAVIS with a public dataset (Intel Research Lab-Seattle) to evaluate its performance. The results are presented in Figure 5b. The loop closure in such a typical office building is 12 cm and is marked as the red line and the yellow line in Figure 5a. In conclusion, the NAVIS mapping result is accurate enough for SOP fingerprint database creating and positioning.

### 2.4. Fingerprint Map Creation

**Figure 6:**
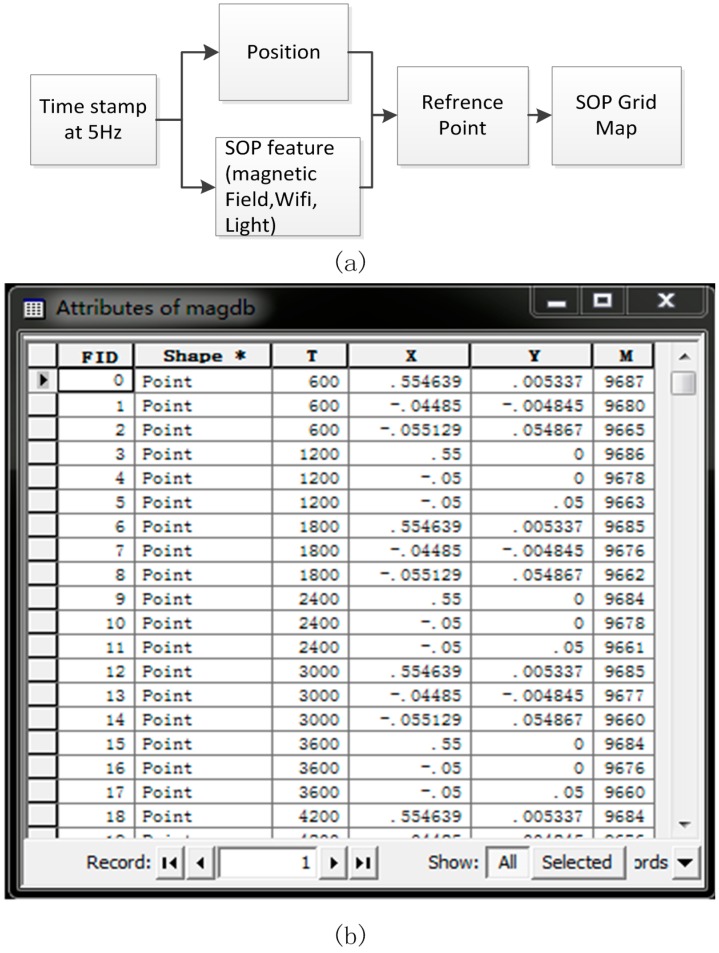
(**a**) Data process workflow of fingerprint map creation; (**b**) an example of a result from the position-magnetic field pairs list from the data process.

In order to acquire the parameters of the SOP fingerprint feature, the SOP sensors are installed on the platform. As shown in Figure 6a, the SOP information is time-tagged with SLAM time. A list of position-SOP pairs is created for the map database when the NAVIS runs along a trajectory consisting of reference points in the corridor. Finally, the SOP grid map can be interpolated from the reference points using the Inverse Distance Weighted interpolation (IDW) algorithm [20,21] within the indoor map boundary. Figure 6b shows an example of a result from the position-SOP pairs list.

**Figure 7:**
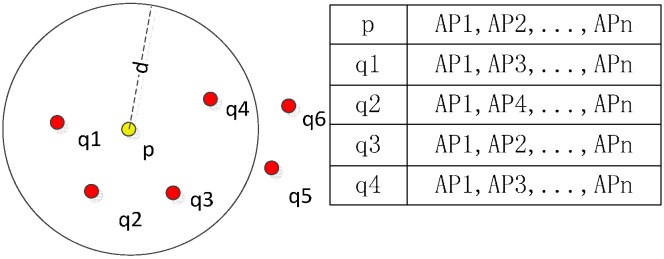
The spatial distance-mean filter for WiFi fingerprint feature information creation.

The fingerprint feature information of magnetic field and light utilised in this paper are normalised intensity data and raw light intensity. Meanwhile, the fingerprint information of WiFi is more sophisticated because there are many WiFi emitters in the FGI main building. Each one transmits signals, and the intensity of signals changes slightly within a short period. A spatial distance-mean filter is adopted on every reference point for acquiring the fingerprint feature information to process the SOP measurements acquired from a moving platform. As shown in Figure 7, the main idea of the spatial filter is to give a certain distance *d* to each reference point *p* (for example, 1 m in this paper). At reference point *p*, *n* WiFi access points (*AP*1, *AP*2, …, *AP*n) are detected, and the mean signal strength of *AP*1 can be calculated with Equation (1) at that reference point:

(1) $$FP\;feature{\left(AP1\right)}_{p}=\frac{1}{n}\overset{n}{\underset{i=1}{\sum }}q_{i}\left(AP1\right)\;when\;Distance\left(p-q_{i}\right)<d$$

### 2.5. Coordinate Reference Rectification

SLAM is a technology of detecting the unknown environment while positioning simultaneously [22,23,24]. It adopts a local coordinate reference on each update cycle, and the orientation of the map depends on the initial position and heading angle of the UGV. The local coordinate reference map should be rectified to a global coordinate reference for further SOP positioning application, and the method applied in this paper is the Four Parameters Similarity Coordinate Transformation (FPSCT), also called two-dimensional Helmert transformation [25]:

(2) $${\left[\begin{array}{c} x\\ y \end{array}\right]}_{global}=(1+m)\left[\begin{array}{cc} \text{cos}\alpha & \text{sin}\alpha \\ -\text{sin}\alpha & \text{cos}\alpha \end{array}\right]({\left[\begin{array}{c} x\\ y \end{array}\right]}_{local}+\left[\begin{array}{c} \text{Δ}x\\ \text{Δ}y \end{array}\right])$$

where Δx and Δy are the displacement of local and global coordinates, respectively, α is the rotation angle and m is the scaling factor.

**Figure 8:**
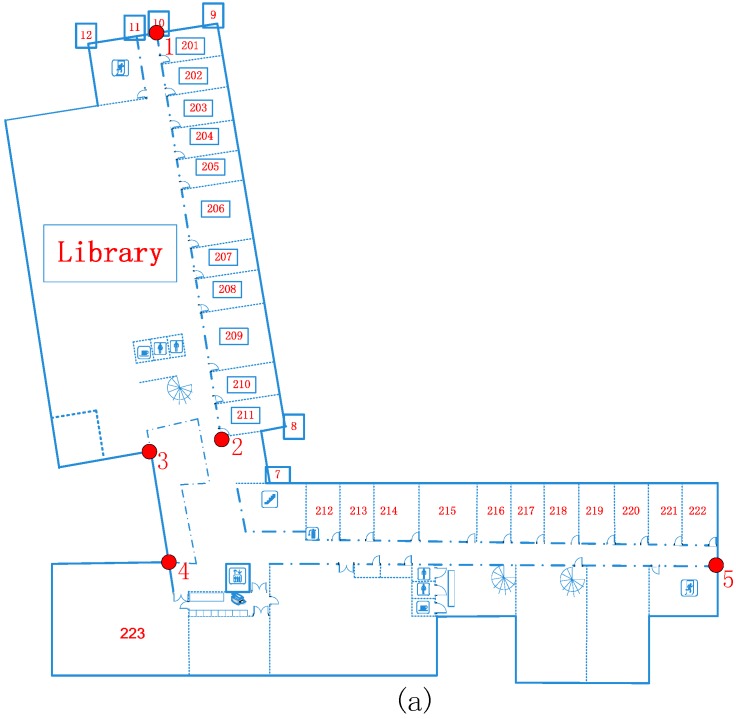

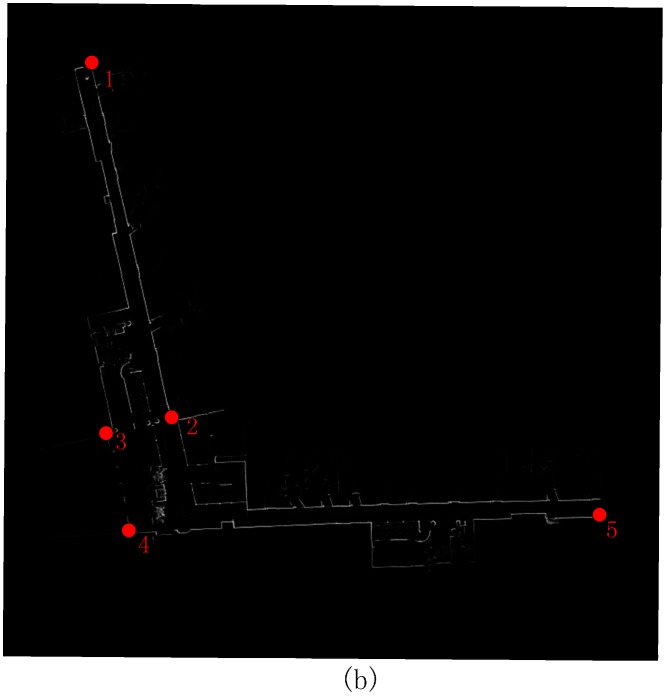
(**a**) The global coordinate map with control points for coordinate rectification; (**b**) example of a local coordinate grid map with control points.

Meanwhile, the four transformation parameters can be calculated with the known control points by the least squares method. The selected control points of the FGI indoor map are shown in Figure 8, which are selected for the transform parameter (Δx, Δy, α, and m) calculation. Finally, the SOP map can be rectified to the global coordinates with Equation (2).

## 3. Tests, Results and Discussion

The field tests were carried out along the corridor at the third floor of the FGI building. To investigate the potential of the proposed method, total nine tests were divided into three groups. Each test lasted for 5 min along the 90 m corridor, and the robot operated at a fixed speed of 0.28 m/s in all tests.

**Figure 9:**
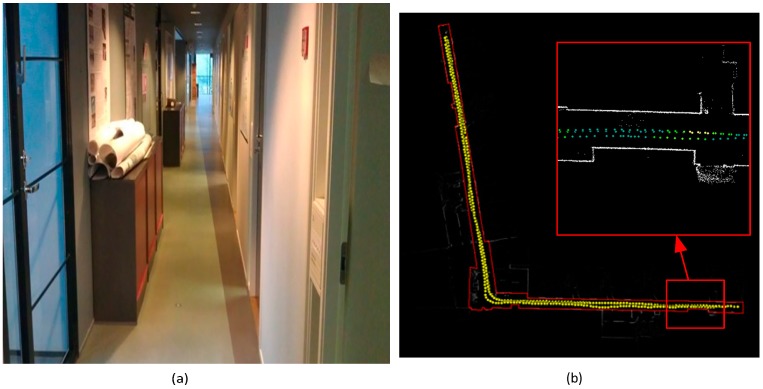
(**a**) Corridor in FGI; (**b**) reference point trajectories collected by NAVIS.

Group 1 (test 1–test 5) was tested for generation of the SOP fingerprint map using magnetic field, light intensity and WiFi RSSI; group 2 (test 6, test 7) was tested for spatial environmental change detection for indoor map updating; and group 3 (test 8, test 9) was tested for SOP environmental change detection for updating the SOP database by turning the light and WiFi APs on and off. Figure 9a,b shows the image of the experiment corridor and a group of reference point trajectories.

### 3.1. SOP Fingerprint Map Generation

**Figure 10:**
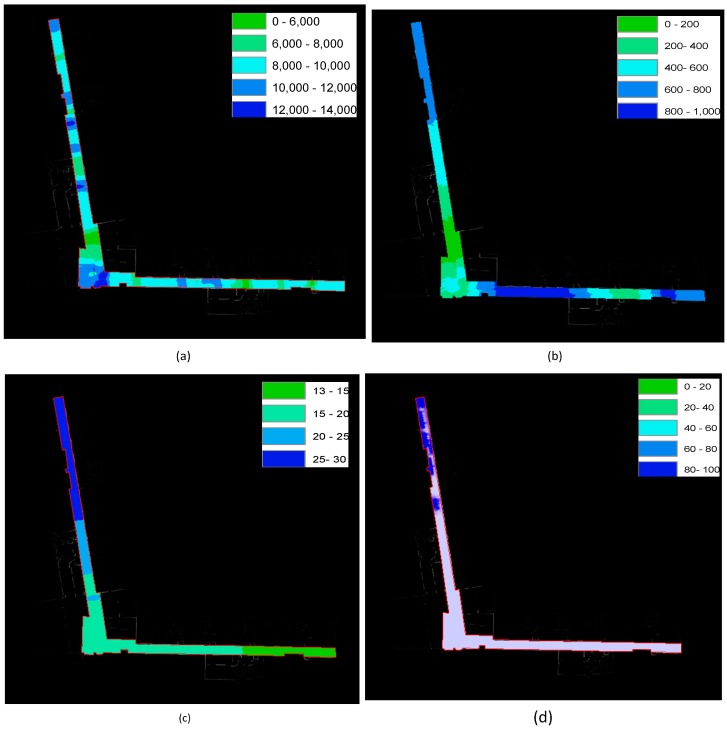
(**a**) Grid map result of magnetic field intensity distribution; (**b**) grid map result of light intensity distribution; (**c**) grid map of WiFi access point number distribution; (**d**) grid map of intensity distribution of one WiFi access point.

As introduced above, multi-SOP sensors can be equipped on the NAVIS platform to acquire related SOP fingerprint feature information. The SOP fingerprint maps are created from the trajectories of group 1. In the trajectories of group 1, there are no obstacles in the corridor, the man-made lights are turned off and all the experimental WiFi APs are turned on. Figure 10a,b shows the grid maps of the discovered magnetic field and light intensity distribution information, respectively. Figure 10c,d shows the detected WiFi AP numbers at different positions and RSSI distributions of one WiFi AP. The reference point positions coincide well with the background-rectified map. The reference points are denser compared with traditional fingerprint creating methods.

### 3.2. Map Variation Detection

The spatial structure of the indoor environment is another important issue for guaranteeing the reliability and positioning accuracy of the indoor positioning system [26]. However, variation in the indoor environment is more frequent than in the outdoor environment. As we know, the variation in the indoor environment may result in a change in SOP pattern. For example, an aquarium may change the indoor map and also attenuate the fingerprint signal like WiFi and Bluetooth; a bulky metal container may distort the magnetic field. Thereby, it is a challenge to detect the variation in the spatial structure and update the indoor map in time.

**Figure 11:**
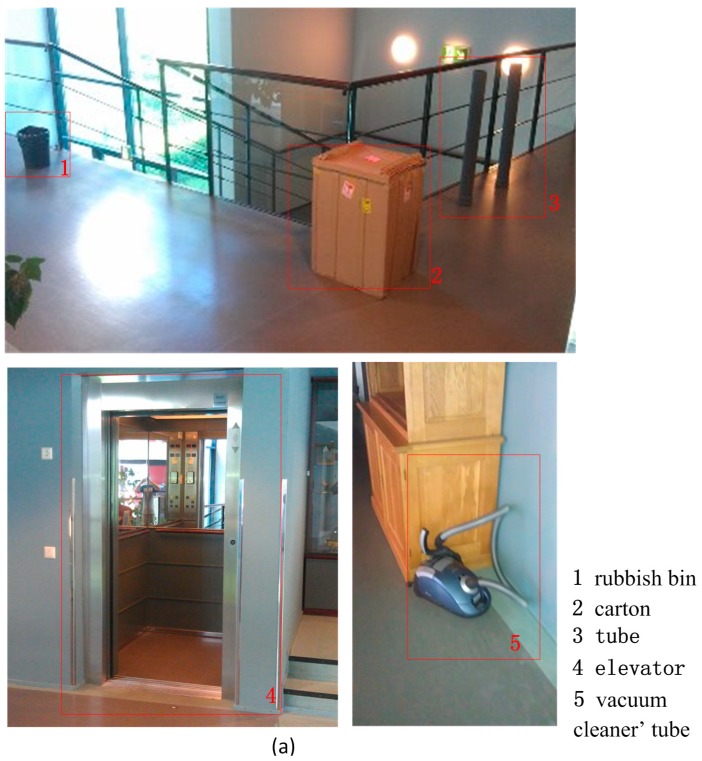

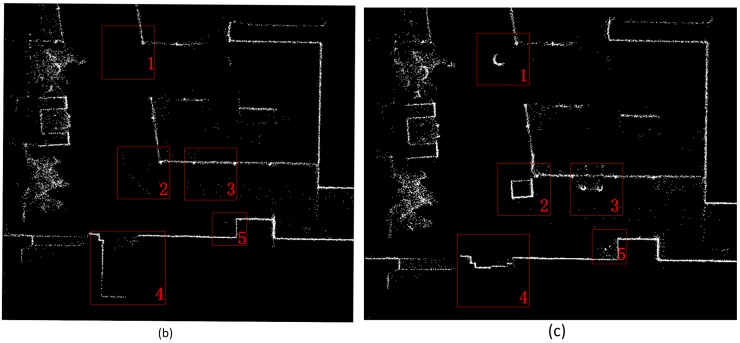
(**a**) Real objects in the corridor; (**b**) Grid map of corridor turn without obstacles; (**c**) Grid map of corridor turn with obstacles.

Figure 11 shows the compared grid maps of the experiment corridor of group 2 and group 1 to demonstrate the capability of detecting changes in spatial structure. As presented by the red rectangles in Figure 11b, there were no obstacles in the corridor turn, and the elevator door was opened in the group 1 experiment. Then, the testers placed a rubbish bin, a big carton, two plastic tubes and a vacuum cleaner on the corner and closed the elevator door to simulate environmental layout changes as presented in Figure 11a. From Figure 11c, all placed objects and environmental changes were detected. The summary of the map variation detection is listed in Table 1. It was found that plenty of noises are introduced by adding the tube in the test scene. More noise can be found in vacuum cleaner case. The explanation of the noise generation is that the diameter of the footprint of the laser scanning point is comparable with or even larger than the size of the detected objects. According to the datasheet of the laser scanner, the diameter can be calculated with Equation (3):

Diameter = distance (mm) × 0.015 rad + 8 mm
(3)

The size of the vacuum cleaner’s tube is approximately 6 cm, which is smaller than the footprint size. The detected tube is 9 cm; however, such point clusters are easily recognised as discrete spatial noise and neglected when converting the grid map to a vector map. Based on the observations and analysis, it can be concluded that for indoor mapping updating, the footprint size should be small enough to detect precise spatial change.

**Table 1:** The summary of the accuracy of map variation detection on different object (unit: cm).

	True Size	Measured Size	Error
Rubbish bin	30	28.62	4.6%
Carton	55 × 45	51.41 × 43.38	6.5% × 3.6%
Tube	14	9.5	32.2%
Vacuum cleaner’s tube	6	9	50%

### 3.3. SOP Variation Detection

**Figure 12:**
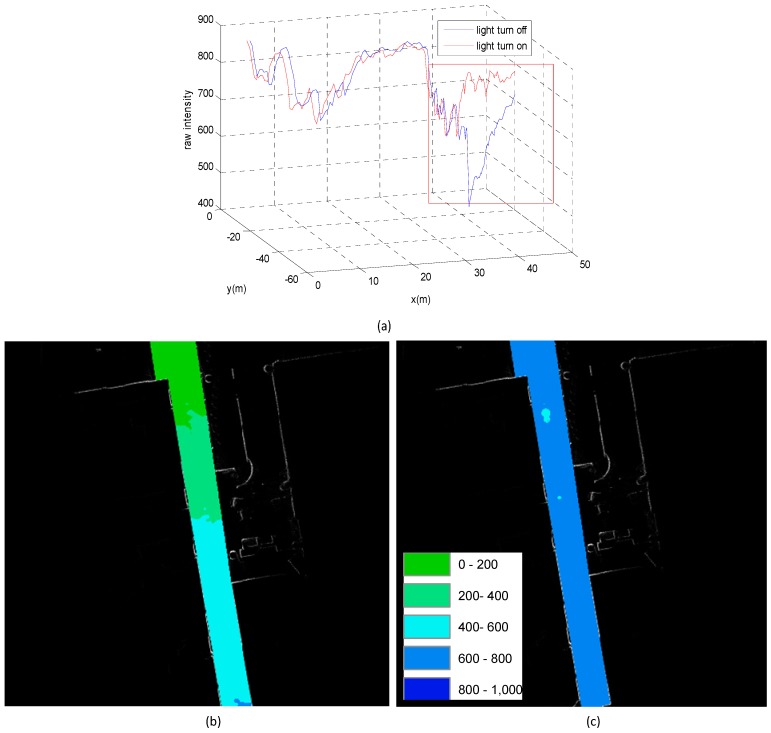
(**a**) 3D plot of light intensity variation detection; (**b**) the light intensity RSSI map with lights turned off; (**c**) the light intensity RSSI map with lights turn on.

It is important to detect SOP variation and update the fingerprint database to assure the reliability of the SOP fingerprint database for the fingerprinting method. In this research, the tester simulated the variation of light and WiFi by turning on/off the devices (light and WiFi emitters) to evaluate the SOP variation detection capability of the proposed system in the group 3 tests. Figure 12 illustrates the results of the light variation detection. In Figure 12a, the red rectangle area represents the variation in light intensity when the lights were turned off (blue line) and turned on (red line). There were several lights equally distributed along the corridor, and similar peaks could be found in the red line. The locations of the peaks coincided with the geospatial distribution of the man-made lights. Figure 12b,c shows the compared light strength RSSI map for different light situations. Then, three WiFi APs were turned off for group 3 located at the beginning of corridor in the junction of two corridors and at the end of the corridor to test the detection of WiFi variation. Such changes could be detected during the group 3 tests.

### 3.4. Evaluation of Indoor Positioning Based on Miscellaneous SOP

A preliminary test has been carried out to evaluate how miscellaneous SOP sources improve the fingerprinting method to evaluate the scalability of the proposed system. An un-optimised Weight Quick Selection (WQS) indoor positioning algorithm is applied for evaluation, which could be considered as a variation of the traditional K-Nearest Neighbours (KNN) algorithm [27,28]. The pseudo-code of the algorithm is shown in Algorithm 1.

**Algorithm 1.** Pseudo-code of the WQS algorithm based on miscellaneous SOPs **Requires**: 1. Fingerprint database F_pdb_ = $\left(P{<x,y,soplist,w>}_{1},\;P{<x,y,soplist,w>}_{2},\ldots ,P{<x,y,soplist,w>}_{n}\right)$;    2. Fingerprint information at unknown position Fp_x_ = (${sop}_{1},{sop}_{2},\ldots ,{sop}_{n}$); **Setting**: ${\text{δ}}_{1},{\text{δ}}_{2}\ldots {\text{δ}}_{m}$ (thresholds) for each type of SOP **for** each sop measurement in Fingerprint Fp_x_   **if**
$abs({sop}_{i}-{soplist}_{j})<\;{\text{δ}}_{1}$   $w_{j}=\;w_{j}+1$ (weight increased only when the sop measurement follows the weight δ-selecting criteria )   **end for** **end for** **return**
$P_{\text{mean}}=\frac{\left(w_{m1}+w_{m2}\right)*P_{1}+\left(w_{m2}+w_{m3}\right)*P_{2}+\left(w_{m1}+w_{m3}\right)*P_{3}}{2*(w_{m1}+w_{m2}+w_{m3})},$which is the mean position of the three positions with the maximum weight $w_{max}$

**Figure 13:**
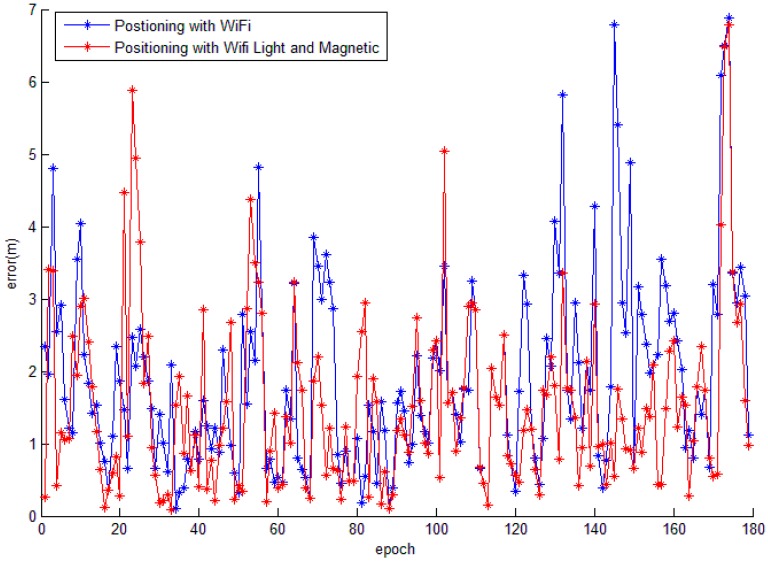
Comparison of positioning errors between single SOP source and miscellaneous SOP sources

An un-optimised and empirical weight δ-selecting criteria is adopted in this research, and the δ values for light, magnetic field and WiFi are 50, 100 and 5, respectively for the WQS algorithm. The results of the fingerprinting method with a miscellaneous SOP source are presented in Figure 13. The blue line in Figure 13 depicts the positioning error results using WiFi only, and the red line shows the positioning error result of the combined solution which utilises the SOPs of WiFi RSSI, light intensity and magnetic intensity. The positioning errors are calculated with Equation (4):

(4) $$\text{e}=\sqrt{{\left(\text{x}-\text{X}\right)}^{2}+{\left(\text{y}-\text{Y}\right)}^{2}}$$

where (x,y) is the estimated position and (X,Y) is the true position. As shown in Figure 13 and Table 2, several conclusions can be drawn: (1) with the proposed UGV mobile platform, the collected SOP fingerprint is comparable with the traditional manual method (1.89 m *vs*. 1.87 m); (2) approximately 80% of the positioning results have better position accuracy than 3 m with WiFi only; meanwhile, when miscellaneous SOP sources are utilised, an un-optimal WQS algorithm can mitigate the positioning mean error by 19% and enhance 10.5% of the positioning result to have a better position accuracy than within 3 m.

**Table 2:** The positioning error statistics (m).

	RMS Error	Mean Error	Maximum Error	Within 3 m
Positioning with WiFi	2.31	1.89	6.87	80%
Positioning with WiFi, light and magnetic	1.95	1.53	6.78	90.5%

The preliminary tests prove that such fast fingerprint maintenance solution can be employed for the fingerprinting indoor position application and can improve positioning accuracy with miscellaneous SOP sources.

## 4. Conclusions and Future Works

This paper presents a fast method for SOP fingerprint database maintenance for indoor positioning based on the self-developed real-time UGV SLAM platform. Based on the results of field tests, the following conclusions can be drawn: (1) SLAM-enabled UGV is a feasible platform for collecting and updating the SOP fingerprint database with finer sampling points and a larger coverage area in a rapid way to sustain the freshness of the database, which is important for a more realistic indoor positioning application; (2) the spatial environment and its variation could be detected and updated by the NAVIS platform, and the outputs can be used for SOP fingerprint database maintenance and positioning purposes; (3) the SOP fingerprint databases maintained by NAVIS are effective and comparable to the traditional manual method; (4) the platform can be easily extended from a single SOP source to miscellaneous SOP sources to increase positioning accuracy and availability.

Based on current configuration of the platform, more experiments will also be carried out in a dynamic environment, where more pedestrians are moving to simulate the data-collecting scenario in a crowded supermarket to verify the performance of the proposed method in more complex situations. Optimised positioning algorithms based on miscellaneous SOP sources will also be investigated.
